# Global Scientific Discourses on Maternal Nutrition: A Scientometric and Altmetric Exploration into Research Trends and Patterns

**DOI:** 10.12688/f1000research.168615.1

**Published:** 2026-01-09

**Authors:** Samir Kumar Panigrahi, Vijayeta Priyadarshini, Sucharita Pradhan, Sangeeta Behera

**Affiliations:** 1 Department of Library and Information Science, Kalinga Institute of Industrial Technology, Bhubaneswar, Odisha, 751024, India; 2 Department of Home Science, Dhenkanal Mahila Mahavidyalaya & Govt Women's Junior College, Dhenkanal, Odisha, 759001, India; 3 Department of Library and Information Science, Kalinga Institute of Industrial Technology, Bhubaneswar, Odisha, 751024, India; 4 Library, Government Women's College, Puri, Odisha, 752002, India

**Keywords:** Maternal Malnutrition, Pregnancy, Scientometric Analysis, Normalised Citation, Thematic Analysis, Topic Dendogram, Social Media, Altmetrics

## Abstract

**Background:**

The present study is an endeavor to explore the global focus on maternal malnutrition research across the globe. The study underscores the development of maternal malnutrition research across the globe and may give impetus to researchers, policy makers and entrepreneurs for further research in this domain.

**Methods:**

The study is descriptive in nature and used a mixed method approach that includes bibliometrics and altmetrics techniques to explore maternal malnutrition research. The study initiated with systematic data extraction from SCOPUS Database with proper data extraction criteria. The bibliometrics techniques include quantitative and qualitative techniques. The initial part of the study explores the quantitative dimension, including authorship studies, growth rate of publications, country-wise productivity and citation impact across the studied period. The second part of the study is dedicated to trend analysis that reveals the focused areas in malnutrition research. The third part of this study delves into the social impact of the concerned set of literature by analyzing the social media attention score through Altmetrics.

**Results:**

It is found that there is a relatively low rate of growth of publications, with publications published in 2016 being more impactful and a preference for multi-authored publications over single-authored publications. Regional distribution highlights disparities in the research focus. Thematic analysis identified key clusters, such as acute malnutrition and maternal-child health interdependencies, with emerging areas in public health nutrition and epigenetics. Altmetric analysis has shown an active but declining trend in discourse on academic and social media platforms.

**Conclusions:**

Results underscore the need for holistic interventions addressing both nutritional and psychosocial factors during pregnancy to break the intergenerational cycle of poor health and promote better outcomes for mothers and their children.

## Introduction

Nutrition is a dynamic and intricate aspect of human life that extends beyond simple nourishment. It influences every part of our physiological, cognitive, and emotional wellbeing. A mother’s diet during pregnancy is crucial for both maternal and child health. Enhanced physiological needs during pregnancy require adequate energy, macronutrients, and micronutrients for fetal development and maternal health. During pregnancy, a woman’s nutritional needs increase to support the growing baby, maintain her health, and prepare for breastfeeding. Important nutrients, such as folic acid, iron, calcium, and protein, play vital roles in preventing birth defects, supporting brain development, and reducing risks such as preterm birth, low birth weight, long-term health conditions and mortality rates. ^1– 5^

The micronutrient deficiencies of iron, iodine, and folic acidare still a significant issue globally, especially in low-income countries where access to healthcare is poor. ^6– 8^ Such deficiencies lead to many health risks, such as maternal anemia, preterm birth, and neural tube defects. ^9– 12^ Nutrition also affects breastfeeding and lowers milk quality and infant immunity. ^13^ Mothers suffering from undernutrition have an increased risk of hypertension, low birth weight, and infant mortality, creating vicious cycles of poor health. ^14^

Recent studies have also emphasized how maternal stress interacts with nutrition. Stress may have an impact on appetite, metabolism, and absorption of nutrients, leading to long-term health consequences for the child. ^15– 24^ This interaction embodies developmental plasticity, in which the prenatal period has a lasting impact on lifelong health.

The period of pregnancy is a crucial time for interventions, but most of the time, psychosocial issues such as stress, which can have a direct impact on dietary recommendations and utilization of nutrients by the human body, are ignored. There should be an integrated approach that combines nutritional education, access to healthcare, and stress management, which may enhance the health of mothers and children. Ensuring a balanced diet, proper supplementation, and access to healthcare can significantly improve outcomes for both mothers and babies, laying the foundation for a healthier future.

## Materials and methods

**Figure 1.**
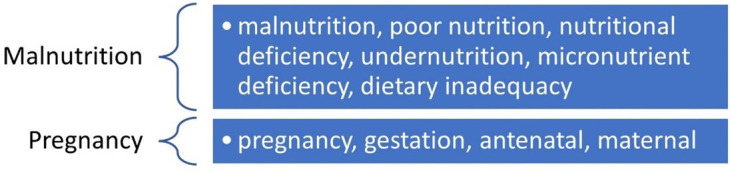
Keywords.

The study initially divided the keywords into two groups such as “Malnutrition” and “Pregnancy”. ^25^ All possible synonymous keywords related to these two groups were collected and connected with the Boolean Operator to create a relevant search string to obtain an exhaustive list of literature related to “Maternal Malnutrition in the database. Figure 1 shows a list of the keywords related to Malnutrition and Pregnancy.

**Figure 2.**
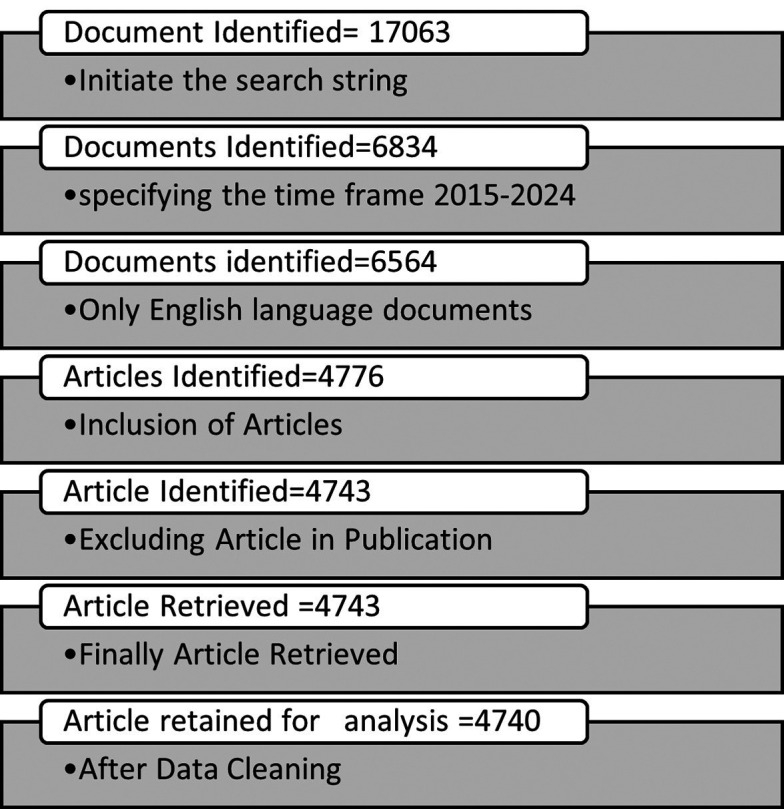
Search technique in SCOPUS database.

By using the search string, the study conducted a literature search in SCOPUS database. Scopus database is a wide-ranging platform that has been argued to be universally popular as a tool for research publication coverage, quality, and relevance in scientometric analysis. ^26– 30^ The documents identified after initial search in SCOPUS database have been filtered out by using various inclusion and exclusion criteria. The documents published only between year 2015 to 2024 have been identified, only English language documents included and the publications type were selected as “Article” to maintain the homogeneity of publications and the “Article in Publication” have been excluded. After using these exclusion and inclusion criteria a total of 4743 articles were selected to export in CSV file format by choosing the all-bibliographic details for the 4743 articles. After exporting the CSV file manual data cleaning work is done and it was found that a total of three number of works did not have the authors name hence these works were deleted and a final 4740 articles were retained for further analysis. Figure 2 shows the inclusion and exclusion criteria and the available number of results from each step of the search techniques.

It was observed that the articles were authored by either a single author or multiple authors. To study the authorship pattern in the concerned set of literature, the publications were divided into two groups: single-author publications, publications with a single author and multiple author publications, and publications with two or more authors. The year-wise distribution of the number of publications, citations, and average citations per publication across the year is presented in Table 1.

**Table 1.** Year wise distribution of publications, citations and average citation.

Year of publication	Single author			Multiple author		
	No. of publications	No. of citations	Average citations	No. of publications	No. of citations	Average citations
**2015**	22	223	10.136	322	21901	68.016
**2016**	24	702	29.250	336	21906	65.196
**2017**	20	586	29.300	378	16138	42.693
**2018**	10	155	15.500	433	11681	26.977
**2019**	16	205	12.813	456	8231	18.050
**2020**	17	241	14.176	523	12593	24.078
**2021**	19	81	4.263	527	5388	10.224
**2022**	16	53	3.313	540	2831	5.243
**2023**	15	16	1.067	527	1432	2.717
**2024**	14	1	0.071	525	1020	1.943

The authors tried to normalize the citations across publications published across the studied period. A suitable year can be defined as the number of years a publication is available for citations. For instance, the citable year for an article published in 2015 is 10 years, for an article published in 2016 is nine years, and for an article published in 2024 is one year, as we have taken the data in 2024. The Total citable year is defined as the total number of available citable years for all articles published in a given year; this is calculated by multiplying the number of publications by the citable year. Normalised citation is a measure of how efficiently the publication of a given year accrues citations in proportion to its outflow time. There is a normalization done, which helps mitigate the bias of older publications getting time to accrue more citations than newer ones. The formula used to calculate the Normalised citation is given in Equation 1.

$$\text{Normalised Citation}=\frac{\text{Citations recived in}\;a\;\text{particular year}\;}{\left(\text{Citable year for}\;a\;\text{particular year}\right)*\left(\text{Number of publications in that year}\right)}$$
(Equation 1)

Table 2 represents the Normalised citations received across each studied period. It can be observed that the Normalised citation score is highest for 2015, 2016, and 2017, of which 2016 topped the list, which shows that articles published during 2016 are more impactful than articles published during any other period.

**Table 2.** Year wise normalised citation.

Year of publication	No. of publications	No. of citations	Citable years	Total citable year	Normalised citation
2015	344	22124	10	3440	6.431
2016	360	22608	9	3240	6.978
2017	398	16724	8	3184	5.253
2018	443	11836	7	3101	3.817
2019	472	8436	6	2832	2.979
2020	540	12834	5	2700	4.753
2021	546	5469	4	2184	2.504
2022	556	2884	3	1668	1.729
2023	542	1448	2	1084	1.336
2024	539	1021	1	539	1.894

### Relative Growth Rate (RGR) and Doubling Time (DT)

RGR and DT provide an understanding of the growth rate and dynamicity of publications. Higher RGR values tend to increase the growth rate of publications, whereas lower DT values indicate rapid growth in publications. The doubling time (DT) is defined as the time required to double the total number of articles. The present study used the formulas mentioned in Equations 2 and 3 to calculate the RGR and DT. ^31– 33^ In the calculation of the RGR, *W1* is the natural logarithm of the initial number of papers, *W2* is the natural logarithm of the final number of papers, *T1* is the initial year, and *T2* is the final year. DT is inversely proportional to the RGR. In Equation 3, 0.693 is the natural logarithm of the two variables.

$$\mathrm{RGR}=\frac{\left(W2-W1\right)}{\left(T2-T1\right)}$$
(Equation 2)

$$\mathrm{DT}=\frac{0.693}{\mathrm{RGR}}$$
(Equation 3)

Table 3 demonstrates the year-wise trends in the Relative Growth Rate (RGR) and Doubling Time (DT) for publications between 2015 and 2024. The total number of publications has grown significantly from 344 in 2015 to 4,740 in 2024. During the emergent phase (2016–2018), RGR was high (0.716 in 2016) and declined to 0.338 by 2018, whereas DT increased from 0.968 to 2.051 years. This indicates the rapid growth that is typical of a nascent research field. In the transition phase (2019–2021), RGR steadily decreased from 0.267 to 0.194 and DT increased from 2.600 to 3.581 years, reflecting slower growth as the field began to stabilize. The mature phase (2022–2024) showed continued declines in RGR (0.165 in 2022 to 0.121 in 2024) and steeper increases in DT (4.205–5.741 years), suggesting stabilization as publication growth slowed. These trends highlight the progression of the domain from a rapid initial growth to a stable and consolidated stage, following the typical lifecycle of a research field.

**Table 3.** Year wise RGR and DT.

Year of publication	Total publications	Cumulative	log W1	log W2	RGR	Doubling time
**2015**	344	344	0	5.841		
**2016**	360	704	5.841	6.557	0.716	0.968
**2017**	398	1102	6.557	7.005	0.448	1.547
**2018**	443	1545	7.005	7.343	0.338	2.051
**2019**	472	2017	7.343	7.609	0.267	2.600
**2020**	540	2557	7.609	7.847	0.237	2.921
**2021**	546	3103	7.847	8.040	0.194	3.581
**2022**	556	3659	8.040	8.205	0.165	4.205
**2023**	542	4201	8.205	8.343	0.138	5.017
**2024**	539	4740	8.343	8.464	0.121	5.741

### Country’s scientific production

**Figure 3.**
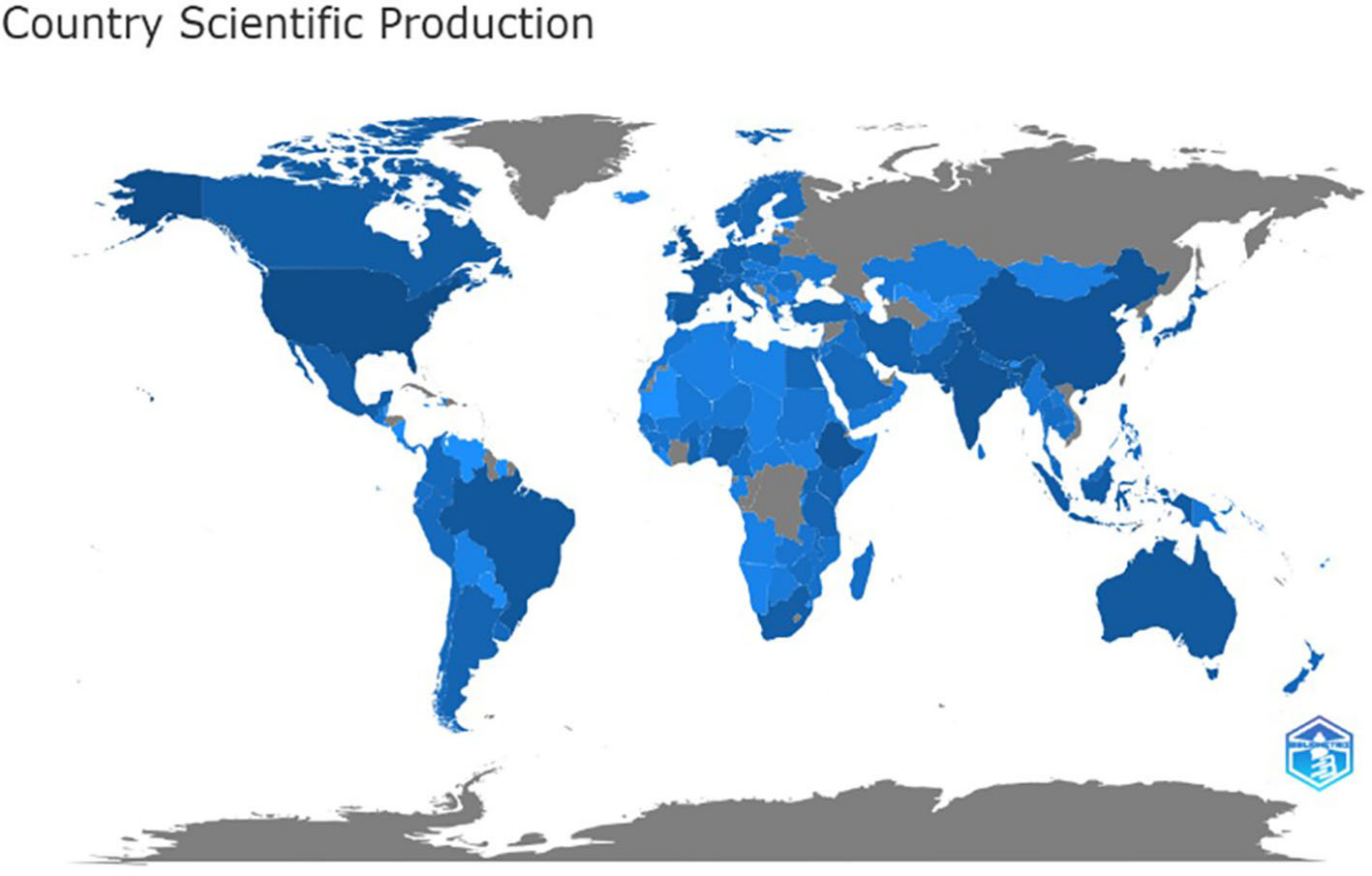
World wide distribution of publications.

The global distribution of literature production is depicted in Figure 3, with the intensity of deep blue indicating higher publication output from specific regions. Regional contributions highlight diverse scientific engagement across the globe. In Latin America and the Caribbean, Brazil has contributed the most, followed by Mexico. Ethiopia and South Africa are the primary contributors to Sub-Saharan Africa, while India and Pakistan dominate South Asia. Iran and Saudi Arabia represent the Middle East and North Africa, respectively, and the USA and Canada lead in North America. In East Asia and the Pacific, China tops the list, followed by Australia, while the UK and Spain are the major contributors in Europe and Central Asia. Globally, the USA has emerged as the top contributor, followed by China, India, Ethiopia, and the UK, underscoring the geographic breadth of research in the domain.

**Figure 4.**
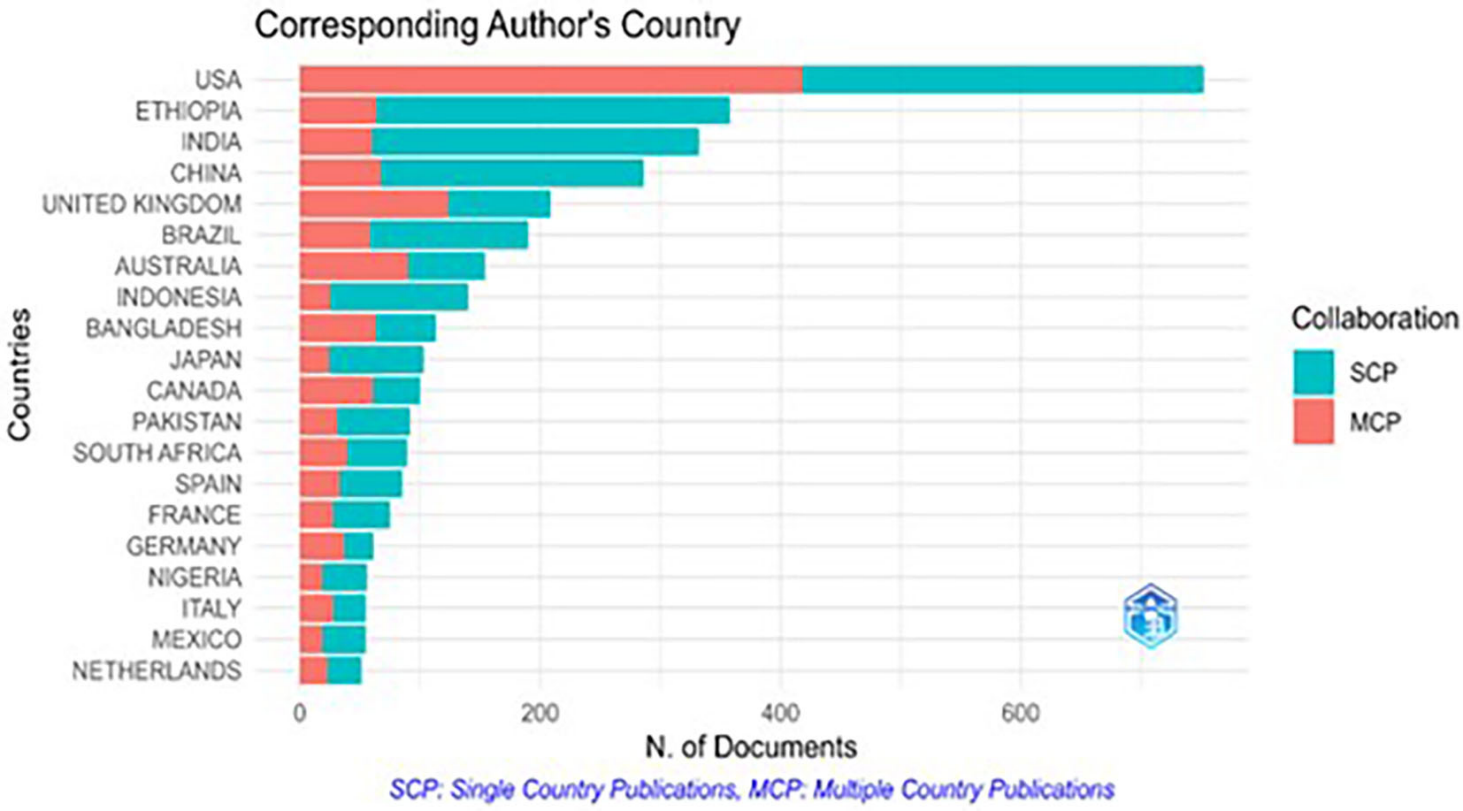
Corresponding author’s country.

The corresponding authors, typically the principal investigators or senior researchers, ensure the accuracy and integrity of a study. Publications are categorized as single-country publications (SCP), involving authors from a single country, or multiple-country publications (MCP), where authors collaborate across nations. Figure 4 illustrates the distribution of the corresponding authors by country, along with the MCP and SCP contributions. The USA leads the corresponding authorship, followed by Ethiopia, India, and the UK. Notably, MCP accounts for over 50% of the publications with corresponding USA-based authors, indicating a high level of international collaboration. In contrast, Ethiopia and India show MCP percentages below 20%, reflecting a stronger focus on local collaboration. Countries such as the UK, Australia, and Bangladesh also exhibit high MCP proportions, surpassing 50%, suggesting a significant emphasis on global research partnerships.

### Cluster analysis and detailed thematic interpretations by using topic dendrogram

**Figure 5.**
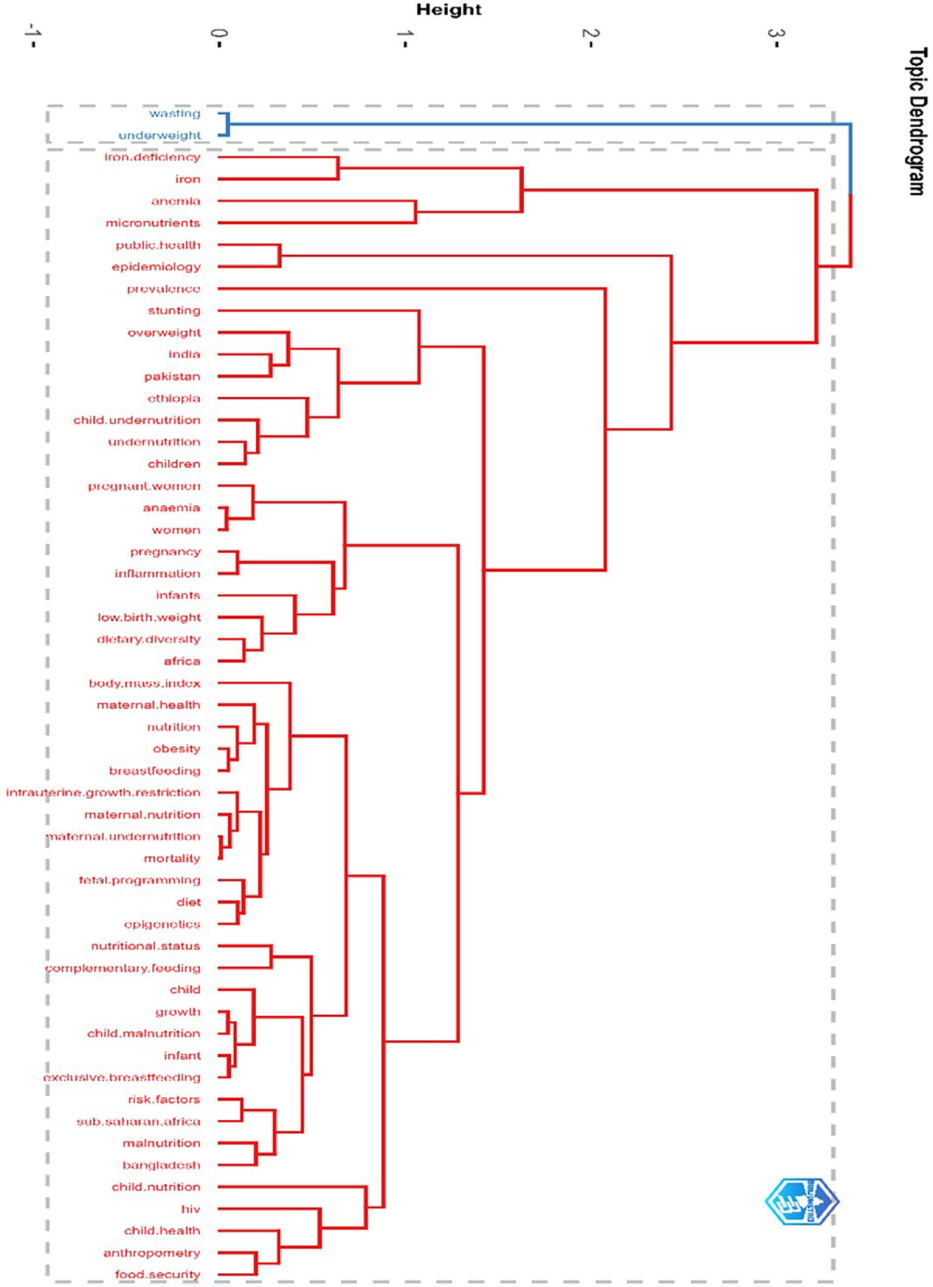
Cluster analysis and detailed thematic interpretations by using topic dendrogram.

The topic dendrogram in Figure 5 displays the hierarchical relationships among the various research themes. A dendrogram was generated by clustering the authors’ keywords using Multiple Correspondence Analysis. Concepts are connected through a hierarchical structure, forming a cluster according to the similarity, and connecting with another cluster through the axis. The shorter axis shows a narrower relationship, and the larger branches show a broader relationship. The vertical axis shows the similarities and dissimilarities among topics. The larger vertical axis shows the distance relationship between concepts, and vice versa. In this case, the focus lies on nutrition, malnutrition, maternal-child health **,** and related public health themes. The vertical axis of the dendrogram lists the key research terms, whereas the horizontal axis quantifies the **degree of similarity or dissimilarity** between these terms. Terms that merge at lower heights exhibit close conceptual relationships, whereas those connected at greater distances signify broader, less direct associations.

### Thematic map

**Figure 6.**
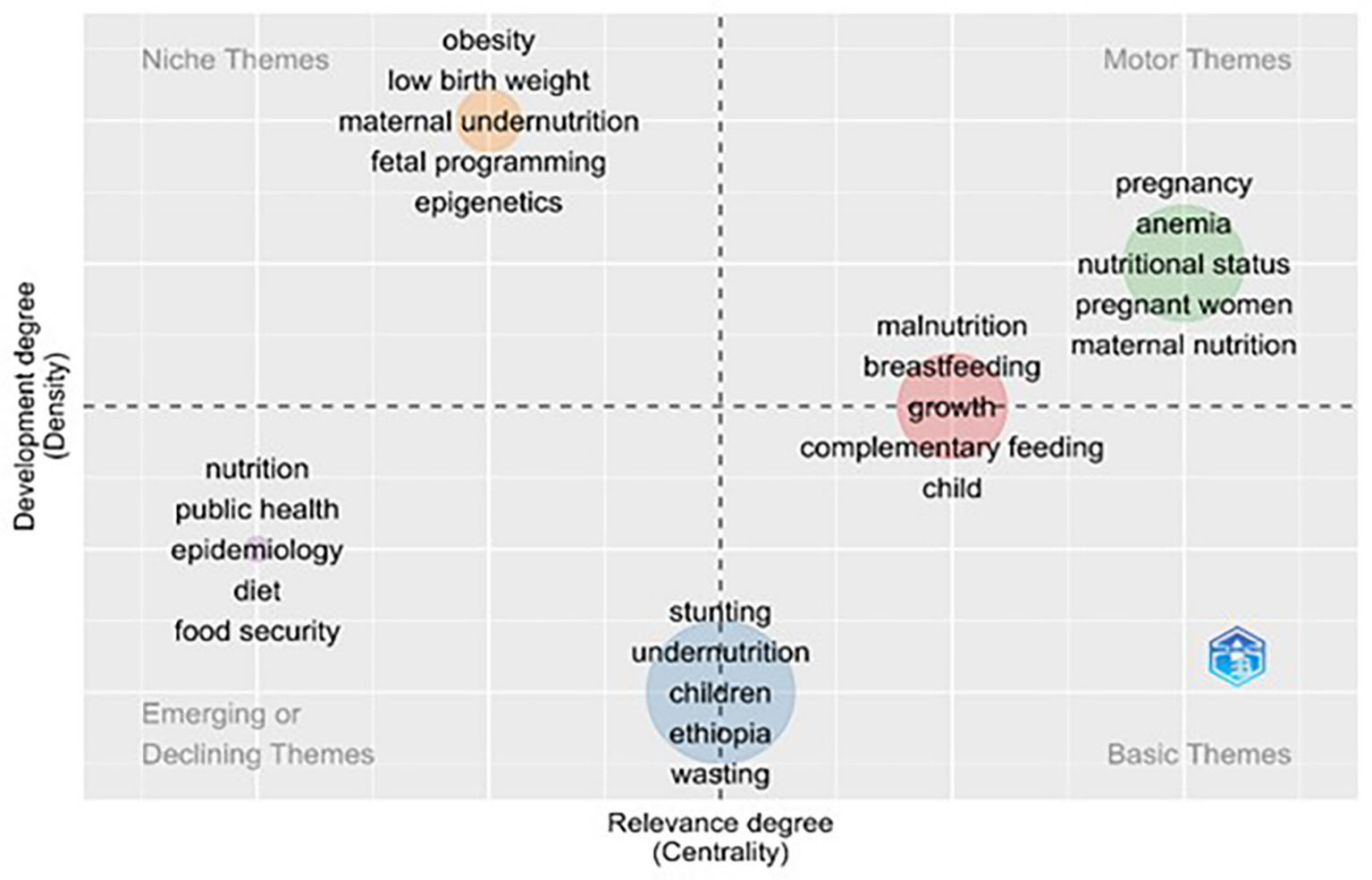
Thematic evolution using author’s keywords.

The four-quadrant approach of the thematic map in biblioshiny provides the idea of thematic areas of research in the concerned domain. Four thematic areas were embedded in the two-dimensional Figure as shown in Figure 6. The Horizontal axis shows the density, which indicates the level of development of the thematic areas compared to the internal association of keywords. The second aspect is centrality, which measures the importance of the thematic area by means of an external association among the keywords. ^34^ Centrality represents the volume of citations associated with the theme, and density represents the number of publications associated with theme. ^29^ The upper-right side of the quadrant is the motor theme, which represents the central and developed areas of the domain. The Upper left quadrant is the Niche Theme, which represents very specialized areas of the domain; the lower left quadrant of the Figure represents the Emerging or Declining theme, which represents the emerging or declining areas of the domain; and the lower right quadrant represents the Basic Theme, which represents the underlying areas of research that are regarded as the base for research development. ^35, 36^

A thematic map is shown in Figure 6, which was created using the keywords of the authors as inputs. The map was generated with specific parameters: a minimum cluster frequency of 5 per thousand, 250 words, 5 labels, and a label size of 0.35. Its thematic map is an informative and insightful visualization based on the authors’ keywords, showing which key themes they refer to and how they are related. The thematic map provided a comprehensive analysis of research trends and themes in the domain of maternal nutrition. This map, structured into four quadrants—Motor Themes, Niche Themes, Emerging or Declining Themes, and Basic Themes—illustrates the density and centrality of various thematic areas, based on scientometric data. Below is a detailed interpretation of each quadrant and the identified clusters.

### Altmetrics approach

The growing importance of social media has ignited scholarly communication in several ways. In this regard, alternative metrics or altmetrics provide a better understanding of public discourse on a particular topic. Traditional citation-based metrics limited their scope to measure the academic impact of scholarly output, but the alternative metrics empower us to capture and understand the social impact of scholarly output. Altmetrics measures scholarly output on various social media platforms. The altmetrics attention score (AAS) on various platforms gives us the idea of how a particular topic is discussed among the public, policymakers, and other stakeholders. In other words, altmetrics empowers us to study the real-world impact of scholarly output in real time scenario. ^37, 38^

**Figure 7.**
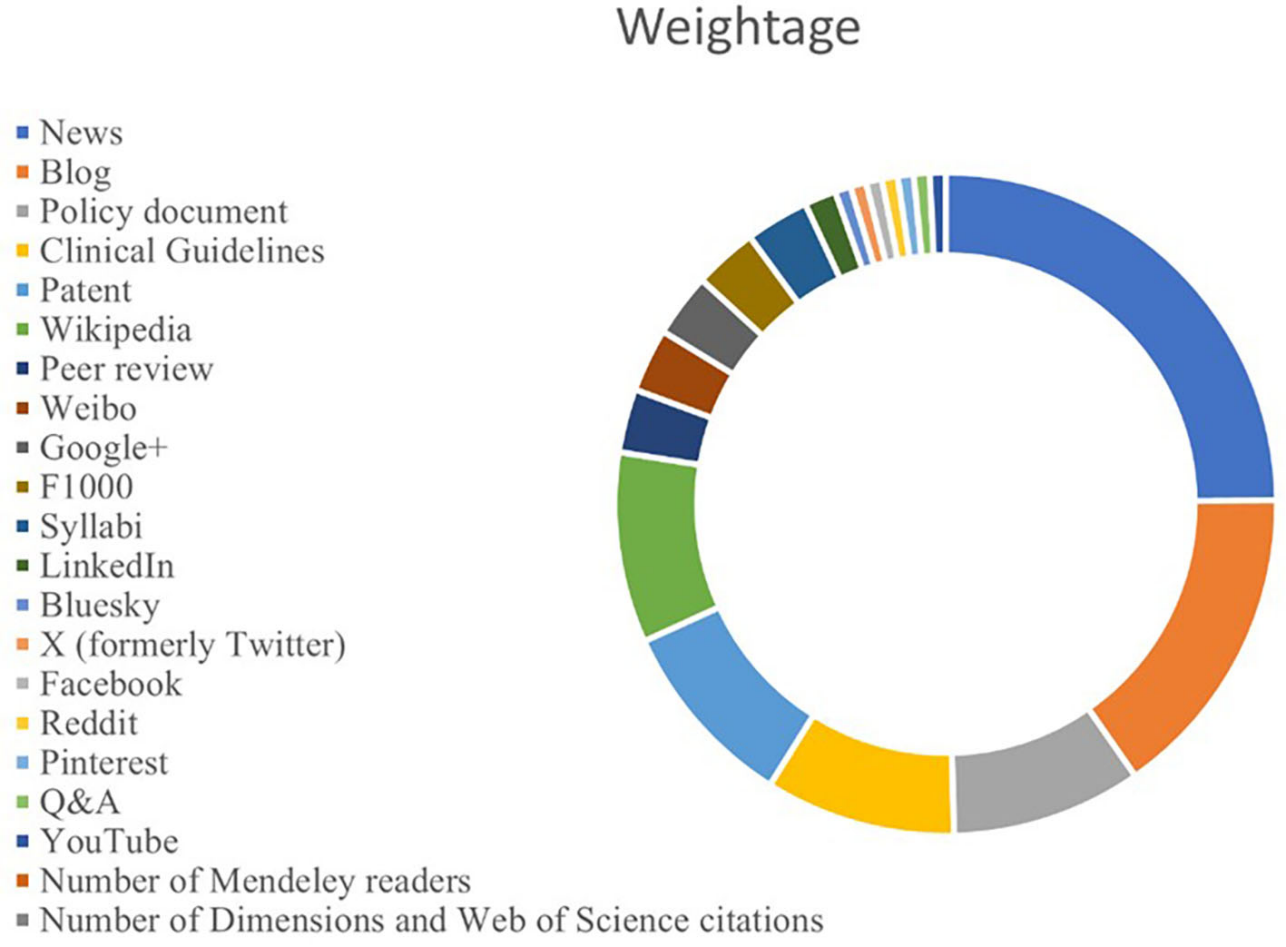
Weightage of platforms.

The study used altmetrics data from altmetrics.com through an API call on Google Sheets. The overall AAS is the weighted average of the article’s engagement on various social media platforms, calculated using an automated algorithm. ^39^ It can be observed from Figure 7 that, out of all platforms, the maximum weightage has been given to “News” (8) followed by “Blog” (5). Online platforms such as “Policy document”, “Clinical Guidelines”, “Patent, and “Wikipedia” have a weighting of three.

Out of 4741 articles, 4589 articles have a digital object identifier (DOI). The DOI of 4589 articles was processed in Google Sheet, and the overall AAS and the individual attention score of each article across various platforms were retrieved through suitable API calls on Google Sheet. It was found that out of 4589 articles, 2529 had zero overall altmetrics scores. It was also found that a total of 763 such articles have zero overall attention score but have at least one attention score in any one of the platforms, since the overall AAS is the weighted average of the attention received across various platforms. The year-wise individual AAS across each platform of each of the articles across all platforms have been summed up. Table 4 shows the year-wise attention scores of all the articles across various platforms. The Data of Table 4 reveal an evolving pattern of individual AAS across the various platforms. A surge in Mendeley readership can be noticed during the years 2015–2016, but a steady decline in AAS over Mendeley can be noticed from the year 2017 to the recent year, which suggests that the particular topic has gained saturated attention or a shift in academic focus during this period. A significantly high score on Blogs and Twitter suggests continuing public discourse on the topic of concern. However, the AAS on Blogs and Twitter can be noticed during the year 2018, 2021 and 2024, which suggests that research conducted during this period gathers more public interest. The AAS on News platform was highest during 2018 and then showed a declining trend, which suggests that research during the year 2018 had potential mainstream attention. The score on policy documents was high during the initial period, which suggests that research conducted during the earlier period was considered for policy discussions, and the gradual declining trend on social media platforms such as Facebook suggests that research during the earlier period attracts more informal attention to social media sites like Facebook. An immediate spike in Wikipedia mentions can be noticed in 2016, but a declining trend can be noticed afterward, which indicates reduced public references or edits related to the topic. A high score on patents can be noticed during the early period, but a declining trend can be noticed afterward, which suggests that research during the earlier period was used to create innovation or technological development in the concerned field.

**Table 4.** Year-wise distribution of individual AAS.

Year platforms	2015	2016	2017	2018	2019	2020	2021	2022	2023	2024
**News**	91	74	66	107	52	69	32	33	30	36
**Blog**	5423	5668	6195	8937	3195	5286	10110	4341	2868	7735
**Policy Documents**	120	93	96	96	44	92	50	20	11	1
**Patents**	13	12	8	6	0	0	0	0	0	0
**Wikipedia**	692	1552	54	24	25	6	10	10	1	3
**Weibo**	1	0	0	0	0	0	0	0	0	0
**Bluesky**	0	4	0	3	2	0	3	1	4	5
**Twitter**	3007	2512	4145	6417	2211	3410	4483	3227	1946	6538
**Facebook**	262	160	276	161	72	46	50	38	31	35
**Reddit**	6	4	20	11	9	4	8	6	3	2
**Q&A**	0	0	0	0	1	0	0	0	0	0
**Mendeley**	52458	59737	58961	53091	49812	54682	38781	26427	17332	14219

## Discussion

The observed trends in Relative Growth Rate (RGR) and Doubling Time (DT) are indicative of the evolutionary trajectory of the research field. During the starting years (2016–2018), it was observed that the RGR was higher and the DT was lower, which indicates rapid progress in the research field of maternal malnutrition. This implies that research interest was rising, probably because of emerging issues and ongoing studies in new research areas. However, from 2019 onwards, a gradual decline in RGR and an increase in DT were observed. This marks a period of slow-pace growth where the research output becomes steady, the domain reaches a more established stage in its lifecycle, wherein growth rates stabilize, and research efforts become more consolidated. This simply indicates that it is the normal course of evolution of a research domain, from explosive growth to stabilizing and levelling off.

Country-wise scientific contributions show variations in research output. The study reveals that the USA has the highest number of multiple-country publications (MCP), implicating its strong research capacity, infrastructure, and effective network of collaboration. Ethiopia and India have lower MCP values, leading to limited international collaborations. Strengthening global partnerships can provide countries with greater access to knowledge, information, funding, and joint research possibilities, which may improve the overall quality and reach of their research.

The cluster analysis presented in the dendrogram shows the key thematic clusters. These clusters focused on nutrition and public health research. The first cluster emphasized acute malnutrition and micronutrient deficiencies. It includes terms such as wasting, underweight, iron deficiency, anemia, and iron and highlights the need for integrated interventions. Cluster 2 focused on epidemiology and public health frameworks, and the key terms included epidemiology, public health, and prevalence. This cluster emphasizes the importance of epidemiological methods that help track malnutrition and identify high-risk groups. Cluster 3, with terms stunting, overweight, India, Pakistan, Ethiopia, and child undernutrition, highlights the double burden of malnutrition, especially in low- and middle-income countries (LMICs). Rapid lifestyle changes and nutrient-poor diets are the main reasons for rising obesity in these regions and the coexistence of undernutrition and overnutrition. Cluster 4 focuses on the link between maternal and child health, and how maternal malnutrition affects poor health outcomes in children. It includes key terms, such as child undernutrition, pregnancy, women, low birth weight, inflammation, infants, anemia, and dietary diversity. Cluster 5 examined anthropometric indicators and maternal-child health metrics. Key terms were BMI, maternal health, nutrition, obesity, breastfeeding, maternal undernutrition, IUGR, fetal programming, and mortality. The cluster stresses the role of maternal BMI in identifying undernutrition and overnutrition, and the long-term effects of maternal nutrition on fetal development and child survival. It also shows the importance of exclusive breastfeeding and overall maternal health in improving children’s well-being. Cluster 6 focused on the systemic determinants of malnutrition. It includes terms such as food security, child health, malnutrition, HIV, and sub-Saharan Africa (SSA). This cluster brings attention to structural and regional issues, especially in malnutrition-prone areas, such as sub-Saharan Africa. These findings collectively emphasize the importance of evidence-based policies and multisectoral collaboration to combat malnutrition on a global scale.

The thematic map divides research related to maternal nutrition into four key areas, based on importance and development. The most critical and well-researched topics (Motor Themes) include pregnancy, anemia, and maternal nutrition, laying the foundation for current policies and programs. Specialized but less central topics (Niche Themes), such as obesity and low birth weight. Emerging or less-developed areas (emerging or declining themes) such as public health and food security represent opportunities for future research. Foundational topics (Basic Themes), including stunting, wasting, and breastfeeding, form the core of foundational research on nutrition and continue to be essential in addressing global malnutrition. This framework highlights the need to integrate established knowledge with emerging research areas, while strengthening basic interventions.

Altmetrics analysis reflects how research is received by a wider public. The AAS reflects the concern domain, which gathers potential public attention on Blogs and Twitter. The higher AAS on Patents and Policy Documents during the earlier period suggests a greater amount of actionable research during the earlier period. The Mendley score suggests that although the concern area of research has achieved saturated attention, research is still in the domain of concern in recent times.

## Conclusion

The analysis of global scientific output and its temporal evolution provides key insights. It was observed that the authorship pattern is gradually shifting toward increased collaboration, as multi-author publications have better citation rates than single-author papers. Normalized citation metrics suggest that articles published in 2016 were most impactful; hence, there is a need to use time-adjusted measures for scientometric analysis. The RGR and DT trends represent a steady decrease in the research growth rate, signifying that the publication landscape is maturing. Country wise contributions highlight that the USA has taken the lead, followed by China and India. Altmetric analysis reflects that research relevance is beyond the traditional citation scenario, with significant engagement in formal academic research as well as in informal public discourse. The findings emphasize the necessity of integrating scientometric and altmetric approaches to capture the multifaceted impact of scientific literature, guiding future research prioritization and policymaking in academia and beyond.

## Data Availability

The data for the present study have been retrieved from the SCOPUS database by using the search string, formulated by combination of keywords with Boolean operators. However the details search procedure have been mentioned in the study in Figure 2.
