# Supplementary material for: Global Scientific Discourses on Maternal Nutrition: A Scientometric and Altmetric Exploration into Research Trends and Patterns
Source: F1000Res. 2026 May 1;15:39. Originally published 2026 Jan 9. [Version 2] doi: 10.12688/f1000research.168615.2 (PMC13150442; doi:10.12688/f1000research.168615.2)
Supplement: Peer review history

# Reviewer report: Reviewer response for version 2

**DOI:** 10.5256/f1000research.197461.r481808

**Published:** 2026-05-06

**Authors:** Suryakanta Jena

**Affiliations:** 1 Utkal University, Bhubaneswar, Odisha, India

This study initiated with a systematic data retrieval process from the Scopus database, which includes all possible datasets related to maternal nutrition during the concerned period. The literature cited in this study is largely relevant to the research. The bibliometric methods and metrics used in this study are appropriate to assess both the quantitative as well as qualitative growth of the concerned literature.

 The initial part of the study focuses on the quantitative growth and citation impact of the selected literature. Further, the study also focuses on the global distribution of the literature. Emphasizing the importance of highly cited papers from the dataset, the study highlights the interest areas in the concerned domain. Cluster analysis and thematic evolution figures help to understand the thematic shifts in the concerned areas of research.

 Lastly, the Altmetrics approach has the potential to showcase the real-time influence and engagement of research papers in the public domain. The discussion and conclusion sections of this study also promptly summarize the research findings and suggest directions for further research. The researcher has also made the data available in a public repository, which reflects the scientific rigor and temperament of the researcher.

 Overall, the holistic approach of this study makes a valuable contribution to the existing literature in the field.

Is the work clearly and accurately presented and does it cite the current literature?

Yes

If applicable, is the statistical analysis and its interpretation appropriate?

Yes

Are all the source data underlying the results available to ensure full reproducibility?

Yes

Is the study design appropriate and is the work technically sound?

Yes

Are the conclusions drawn adequately supported by the results?

Yes

Are sufficient details of methods and analysis provided to allow replication by others?

Yes

Reviewer Expertise:

Bibliometrics, scientometrics, research evaluation, research productivity, citation analysis

I confirm that I have read this submission and believe that I have an appropriate level of expertise to confirm that it is of an acceptable scientific standard.

# Reviewer report: Reviewer response for version 1

**DOI:** 10.5256/f1000research.185805.r460441

**Published:** 2026-03-06

**Authors:** Sourav Bhattacharjee

**Affiliations:** 1 National Institute of Technology, Rourkela, Odisha, India

Strengths

- The study reviews and analyses a large dataset of 4,740 publications and provides a useful overarching view of global research trends in maternal nutrition between 2015 and 2024,
- The combined use of scientometric and altmetric approaches is helpful because it captures both academic impact and broader societal engagement through the research,
- The visual analytical tools (thematic maps, dendrogram clusters, and geographic distribution) bring to the fore newer research themes and patterns of global collaboration.

Weaknesses

- The study findings rely only on the Scopus database, which I am afraid may limit comprehensiveness and exclude relevant literature indexed in other databases.
- The link between bibliometric findings and policy or programmatic implications is currently disjointed and needs further strengthening.
- The interpretation of altmetric results and discussion of the limitations of the study (for example, language restrictions, database bias) could be elaborated to facilitate in-depth analysis.

A summary: 

 This article provides a scientometric and altmetric assessment of global research on maternal malnutrition, based on scientific literature published between 2015 and 2024, using data from the Scopus database. Analysing 4,740 articles, the study critically examines publication trends, authorship patterns, citation impact, geographic distribution, thematic research clusters, and social media engagement through Altmetric Attention Scores. The study finds modest growth in the field, more multi-author collaborations, and sizable geographic disparities in research productivity. Key thematic areas identified include hidden hunger, mother and child health linkages, epidemiology, and systemic determinants such as nutrition and food security. The study findings also bring to the fore the importance of digital and social media in shaping broader engagement with maternal nutrition research and researchers. Overall, the article offers a useful overview of the evolving global research landscape in maternal nutrition. Overall, the article is relevant to researchers and policymakers because it provides a broad overview of how scientific discourse on maternal nutrition has evolved globally, which would allow them to take corrective policy actions or delve deeper into areas requiring more research.

Is the work clearly and accurately presented and does it cite the current literature?

Partly

If applicable, is the statistical analysis and its interpretation appropriate?

Partly

Are all the source data underlying the results available to ensure full reproducibility?

No

Is the study design appropriate and is the work technically sound?

Partly

Are the conclusions drawn adequately supported by the results?

Partly

Are sufficient details of methods and analysis provided to allow replication by others?

No

Reviewer Expertise:

Global public health and nutrition policies

I confirm that I have read this submission and believe that I have an appropriate level of expertise to confirm that it is of an acceptable scientific standard, however I have significant reservations, as outlined above.

# Reviewer report: Reviewer response for version 1

**DOI:** 10.5256/f1000research.185805.r450654

**Published:** 2026-02-10

**Authors:** Guangxin Yue

**Affiliations:** 1 China Academy of Chinese Medical Sciences, Beijing, Beijing, China

This study presents a valuable mixed-methods analysis of global research trends in maternal malnutrition. The bibliometric and altmetric approaches are generally well-executed, and the scope of covering 4,740 publications is commendable. However, several areas require improvement to enhance the scholarly rigor and impact of the work. 

 1.While the literature review adequately covers foundational studies, it would benefit from incorporating more recent high-impact publications (2023-2025).

 2.The methods section would be strengthened by providing greater technical detail:

 Share data-processing scripts and API configurations in a public repository (e.g., GitHub), and append a parameter table detailing clustering algorithms and validation metrics used in the thematic analysis

 3.Deposit anonymized data on platforms like Zenodo or Figshare with a persistent DOI

 4.Supplementing citation metrics with qualitative analysis of highly cited papers

 5.Discussing alternative explanations for Altmetric trends, such as social media platform dynamics or algorithm changes that might influence attention scores.

**Overall Recommended Actions:**

 1.Update literature review with recent high-impact publications

 2.Enhance methodological transparency through supplementary materials

 3.Improve data accessibility following FAIR principles

 4.Strengthen interpretation with multi-faceted analysis of key findings

Is the work clearly and accurately presented and does it cite the current literature?

Partly

If applicable, is the statistical analysis and its interpretation appropriate?

Yes

Are all the source data underlying the results available to ensure full reproducibility?

No

Is the study design appropriate and is the work technically sound?

Yes

Are the conclusions drawn adequately supported by the results?

Partly

Are sufficient details of methods and analysis provided to allow replication by others?

Partly

Reviewer Expertise:

Neuroscience, Psychology(mind and behavior)

I confirm that I have read this submission and believe that I have an appropriate level of expertise to confirm that it is of an acceptable scientific standard, however I have significant reservations, as outlined above.

# Response

**Published:** 2026-03-05

**Authors:** Samir Kumar Panigrahi

**Affiliations:** Library & Information Science, Kalinga Institute of Industrial Technology, Bhubaneswar, Odisha, India

We sincerely thank the reviewer for the constructive comments and valuable suggestions, which have helped us improve the quality, clarity, and transparency of the manuscript. All suggested revisions have been carefully incorporated in the revised version of the manuscript. The detailed responses are provided below.

**Comment 1:** While the literature review adequately covers foundational studies, it would benefit from incorporating more recent high-impact publications (2023-2025).

**Response 1:** Thank you for this helpful suggestion. As per the suggestions some of the high impact publications related to maternal nutrition publications, published period 2023-2025, have been added in appropriate place.

**Comment 2:** The methods section would be strengthened by providing greater technical detail:

 Share data-processing scripts and API configurations in a public repository (e.g., GitHub), and append a parameter table detailing clustering algorithms and validation metrics used in the thematic analysis

**Response 2:** Thank you for this excellent suggestion. In response, a separate subsection “Tools and techniques” have been added under Methodology section, which includes more technical details on the data processing procedures and analytical tools used in the study.

**Comment 3:** Deposit anonymized data on platforms like Zenodo or Figshare with a persistent DOI

**Response 3:** Thank you for this valuable suggestion. To enhance transparency and reproducibility, the dataset used in this study has been anonymized and deposited in the Zenodo repository with a persistent DOI.

**Comment 4:** Supplementing citation metrics with qualitative analysis of highly cited papers

**Response 4:** Thank you for this constructive suggestion. In response, we have added two new subsections under data analysis and interpretation section, which depict the distribution of publications according to the citation category and discussion on top ten most cited publications.

**Comment 5:** Discussing alternative explanations for Altmetric trends, such as social media platform dynamics or algorithm changes that might influence attention scores.

**Response 5:** Thank you for this insightful suggestion. A new subsection titled “Limitations of the Study” has been added to the manuscript. This section discusses potential factors that may influence Altmetric Attention Scores along with other limitations of this study.
